# Human Pegivirus Type 1: A Common Human Virus That Is Beneficial in Immune-Mediated Disease?

**DOI:** 10.3389/fimmu.2022.887760

**Published:** 2022-05-30

**Authors:** Jack T. Stapleton

**Affiliations:** ^1^ Medicine Service, Iowa City Veterans Administration Healthcare, Iowa City, IA, United States; ^2^ Departments of Internal Medicine, Microbiology & Immunology, University of Iowa, Iowa City, IA, United States

**Keywords:** *Pegivirus*, GB virus, immune modulation, viral co-infection, *Flavivirus*

## Abstract

Two groups identified a novel human flavivirus in the mid-1990s. One group named the virus hepatitis G virus (HGV) and the other named it GB Virus type C (GBV-C). Sequence analyses found these two isolates to be the same virus, and subsequent studies found that the virus does not cause hepatitis despite sharing genome organization with hepatitis C virus. Although HGV/GBV-C infection is common and may cause persistent infection in humans, the virus does not appear to directly cause any other known disease state. Thus, the virus was renamed “human pegivirus 1” (HPgV-1) for “persistent G” virus. HPgV-1 is found primarily in lymphocytes and not hepatocytes, and several studies found HPgV-1 infection associated with prolonged survival in people living with HIV. Co-infection of human lymphocytes with HPgV-1 and HIV inhibits HIV replication. Although three viral proteins directly inhibit HIV replication *in vitro*, the major effects of HPgV-1 leading to reduced HIV-related mortality appear to result from a global reduction in immune activation. HPgV-1 specifically interferes with T cell receptor signaling (TCR) by reducing proximal activation of the lymphocyte specific Src kinase LCK. Although TCR signaling is reduced, T cell activation is not abolished and with sufficient stimulus, T cell functions are enabled. Consequently, HPgV-1 is not associated with immune suppression. The HPgV-1 immunomodulatory effects are associated with beneficial outcomes in other diseases including Ebola virus infection and possibly graft-versus-host-disease following stem cell transplantation. Better understanding of HPgV-1 immune escape and mechanisms of inflammation may identify novel therapies for immune-based diseases.

## Introduction and Background

Following the discovery of hepatitis C Virus (HCV), it became clear that there are cases of post-transfusion hepatitis that are not caused by hepatitis B virus (HBV) or HCV (1). Since none of the hepatitis viruses are readily cultivated in laboratory cell culture systems, progress in identifying additional etiologies of post-transfusion hepatitis proved difficult. In the mid-1990s, two companies independently used recently developed molecular methodologies to identify a human RNA virus in samples obtained from individuals with non-A, non-B, non-C post-transfusion hepatitis (2–4). The viral genome sequence and organization shared many similarities with HCV and other members of the *Flaviviridae* (2, 4–6), and one of the groups discovering the virus named it Hepatitis G Virus (HGV) (4), while the other was called GB virus type C (GBV-C) (2). “G.B.” were the initials of a surgeon who developed fulminant hepatitis in the 1960s, and whose serum caused transmissible hepatitis in marmosets [reviewed in (1, 7]. This “GB” serum was passaged in marmosets, and two viruses were identified in these materials that were called GB virus A and B (GBV-A, GBV-B) (3). Using these viral sequences as a guide, PCR methods were developed that identified a human virus and this virus was called GBV-C (2).

The “GB” serum reagents were extensively studied in the 1960s and 70s under the premise that they may contain the elusive cause of post-transfusion hepatitis, which ultimately proved to be HCV and not the GB agent (8–10). Following identification of HGV/GBV-C, virologic and serologic studies found that this virus is common in humans and that infection correlates with exposure to blood-born and sexually transmitted infections (4, 11–23). The related GBV-A and GBV-B proved to be non-human primate viruses, and there is no evidence that the surgeon “GB” was infected with GBV-C (reviewed in (7, 24). Further, numerous studies demonstrated that HGV is not specifically associated with acute or chronic hepatitis (14, 21, 25–28). In the majority of infections viremia persists for more than one year and may persist for decades in a subset of individuals (17, 18, 29). The underlying host factor(s) responsible for viral clearance remain unknown.

Since the virus does not appear to cause hepatitis, and there is no evidence that the surgeon G.B. was infected with this virus, the two names describing this virus were not logical. A 2011 proposal to assign the “GB viruses” into a novel genus named “*Pegivirus*” this was accepted by the International Committee on the Taxonomy of Viruses (ICTV) in 2013 (7, 30). *Pegiviruses* were comprised of viruses that demonstrated distant sequence relatedness to other members of the family *Flaviviridae* based on phylogenetic alignments of the RNA dependent, RNA polymerase. In addition to having a distinct phylogenetic position most closely related with the *hepaciviruses* within the *Flaviviridae*, *pegiviruses* differ from other hepaciviruses in genome organization features including an apparent lack of a nucleocapsid protein, a different internal ribosomal entry site (IRES) structure in the 5’ non-coding genome region, and less predicted glycosylation of the envelope glycoprotein E2 (reviewed in (7). The name “*pegivirus*” was derived from “Pe” for “persistent” and “G” representing the historical naming of the virus as HGV and GBV-C (7).

**Figure 1:**
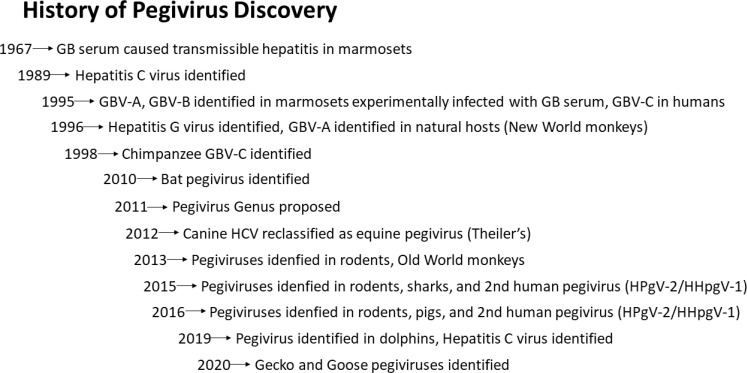
History of *Pegivirus* Discovery.

Although initially found only in primates and bats, the host range of *pegiviruses* has greatly expanded following the advent of next-generation sequencing (31–34). The genus currently has at least 11 host species (Pegivirus A to K) and additional host species are continuing to be identified. Pegivirus A members infect New World primate monkeys (35–37), and bats (38), while members of Pegivirus C infect humans and chimpanzees (2, 4, 39, 40) and other Old World monkeys (41, 42). Human pegivirus is regularly called HPgV-1 although this has not been formally assigned by the ICTV. Excluding HPgV-1 (pegivirus C), species B through J infect a wide range of primates and non-primate hosts including horses, domestic cats, lemurs, bats, dolphins, pigs, and rodent species (31, 38, 43–50). **Figure 1** outlines the chronology of Pegivirus discovery.

Limited information suggests that *pegiviruses* do not transmit readily between host species; however, HPgV-1 can infect chimpanzees (51), and a baboon isolate of Pegivirus C can infect rhesus macaques (42, 52). Remarkably, novel viruses sharing *pegivirus* and *hepacivirus* features are found in non-mammalian hosts including sharks, lizards (Australian gecko), and geese (53–56). The goose pegivirus is reported to replicate in goslings, embryonated goose eggs, and primary goose embryo fibroblasts, and thus is the first *pegivirus* efficiently cultured *in vitro* (55). Until the goose pegivirus was recently described, no *pegivirus* has been described that replicate efficiently in cell culture systems and this has greatly hampered understanding of the biology of this enigmatic virus. The ability of all of the *pegiviruses* to cause persistent infection has not been confirmed.

A second human *pegivirus* closely associated with HCV infection was discovered in 2015 (57, 58). This virus is referred to as HPgV-2 or HHPgV-1, while the original HGV/GBV-C is referred to as HPgV-1. HPgV-2/HHPgV-1 is closely associated with HCV and is a different virus (not a different genotype) than HPgV-1. HPgV-2 is much less prevalent. Although there are few reports elucidating the pathogenic outcomes of this infection, in contrast with HPgV-1, the virus shares some virologic features with HCV including a predicted type IV IRES and highly glycosylated E2 protein (58). Nevertheless, a recent study shows that, like HPgV-1, HPgV-2 appears to be lymphotropic. More study of this virus is needed (59). Since current knowledge related to HPgV-2 is limited, the virus is not in the same phylogenetic lineage as HPgV-1 and is not known to influence other diseases, HPgV-2 will not be discussed further in this review.

HPgV-1 infection occurs globally, and phylogenetic studies identify 7 genotypes that are geographically distributed (60–64). The virus is thought to be ancient based on several lines of evidence. First, genotypes are distributed in patterns that mimic human migration from Africa (7, 61) and the genetic distance between genotypes is similar to that of mitochondrial DNA between peoples of different races (61). South American populations comprised of mixed European and indigenous ancestry have a mixture of genotypes 2 (European/N. American) and 3 (Asian), while indigenous South Americans have genotype 3. Further, the prevalence of the virus in indigenous populations is similar to that of other regions of the world (61, 65–67). Finally, HPgV-1 is closely related to both old world and new world *pegiviruses*, suggesting that the virus was in primates prior to the split of the continents (61). Taken together, it has been suggested that HPgV-1 was present in primordial humans. Of note, recombination between genotypes has been described, which may complicate phylogenetic analyses and interpretation of genotypic distributions (68).

**Figure 2:**
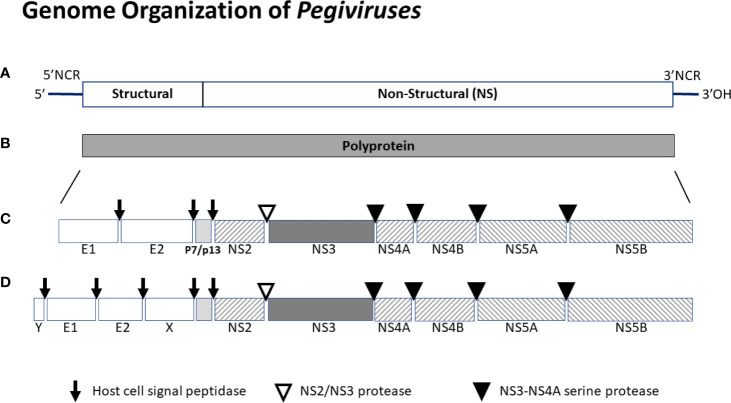
Schematic of the genome organization of *pegiviruses*. *Pegivirus* genomes range from 8.9 to 11.3 kb, and those with longer genomes (e.g. HPgV-2) produce two additional structural proteins (X and Y). The genome **(A)** has a highly structured 5’ noncoding region (NCR) that contains an internal ribosomal entry site directing translation, and a 3’ NCR. The positive sense viral genome is translated and encodes a single polyprotein **(B)** that is co- and post-translationally cleaved into the final viral proteins **(C** or **D)**. Structural proteins are cleaved by host cell signal peptidases. Nonstructural proteins NS2-NS3 are cleaved by the NS2-NS3 autoprotease while NS3 to NS5 proteins are cleaved by the NS3-NS4A protease complex. All *pegiviruses* have two structural proteins (envelope glycoproteins E1 and E2), and those with longer genomes have two additional predicted structural proteins - a basic protein of unknown function upstream of E1 (Y) and some *pegiviruses* contain a predicted glycoprotein downstream of E2 (X). The RNA dependent, RNA polymerase resides in NS5B. The human pegivirus (HPgV-1) genome is approximately 9.4 kb and the final protein organization is shown in the middle panel **(B)**.

HPgV-1 shares many features with HCV. The viral genome is ~ 9.4 kB long, has a positive polarity and serves as the messenger RNA being translated off of its 5’ non-coding IRES (2, 4, 6, 35, 69, 70). The genome includes a long open reading frame that is translated into a C-terminal polyprotein with structural proteins post-translationally processed by cellular signal peptidase, and nonstructural proteins processed by two viral proteases (NS2 and NS3) and the NS3 co-factor (NS4A) (71, 72). A schematic of the viral genome, translation product polyprotein, and final protein composition is shown in **Figure 2**.

## Transmission and Epidemiology

HPgV-1 is transmitted *via* blood exposure, sexual exposure, and from mother to child (11, 12, 17, 21, 73–79). Based on transfusion studies, HPgV-1 infection typically lasts for > 6 months and is cleared in more than 50% of infections within 2 years (12, 18, 80). In a subset of individuals, viremia persists for more than two years, and for longer in some individuals (29, 81). In those who clear viremia, antibodies to the major viral envelope glycoprotein (E2) are usually detected and these antibodies are somewhat, though not completely protective (82–84). Interestingly, more than 90% of viremic individuals do not have detectable E2 antibodies, and no reproducible antibody pattern against any viral protein is elicited, suggesting a mechanism of immune evasion not described for other virus infections (reviewed in (7, 24, 28).

Based on E2 antibody and viremia prevalence studies, HPgV-1 is extremely common (12, 17, 21, 76, 84, 85). Viremia prevalence among healthy blood donors in developed countries ranges from 1.8% to 6% and E2 antibody prevalence ranges from 10 to 15% (7, 29, 67, 84, 86–89). The prevalence of infection in developed countries is estimated to be as high as 20% based on viremia and antibody determination, and infection is two to five times more frequent in developing countries and in individuals with other sexually transmitted or bloodborne infections (7, 29, 67, 84, 86–89). Since antibodies are lost in some individuals over time, prevalence estimates based on viremia and antibody detection likely represent an underestimate of true infection rates. A recent analysis of 79 studies including a meta-analysis of 63 of these found the global prevalence in healthy volunteer blood donors to be 3.1%, with geographic differences ranging from 1.7% in North America to 9.1% in South America (88). Complicating determinations of prevalence, reinfection of people who have lost antibodies is described (29, 90), and a small proportion of viremic individuals have detectable E2 antibodies (29, 91). The mechanisms underlying viral clearance and the combination of viremia with E2 antibodies are unclear.

Of note, no country screens blood donors for HPgV, despite the fact that viremia is common (2, 4). The argument against screening includes the fact that roughly one in 30 donations would need to be discarded in the U.S., greatly reducing blood availability for an infection, HPgV-1, that does not appear to cause significant disease. A recent study found that there are more than 17.9 million units of blood products transfused in the U.S. in 2017 (92), suggesting that approximately 1,000 blood product units containing HPgV-1 are transfused every day in the United States (92).

As noted above, HPgV-1 prevalence is higher among individuals who are highly exposed to bloodborne or sexually transmitted infections compared to healthy blood donors (21, 67, 76, 93, 94). In people living with HIV (PLWH) for example, 39% were viremic and 45% had detectable E2 antibody in the Multicenter AIDS Cohort study group, suggesting near universal infection (29). The proportion of PLWH who cleared viremia over six years of observation also appears to be lower than that of healthy blood donors (29, 81). Viremia rates in PLWH vary between studies, ranging from 15% to 41% [reviewed in (84, 95–97]. Further evidence of reduced clearance in PLWH is drawn from the finding that the ratio of E2 antibody to viremia is higher among people living with HCV infection (98). Given the rate of HPgV-1 viremia in healthy blood donors and the increase prevalence among people with other transmission risks, it appears that perhaps one third of humans experience HPgV-1 infection during their lifetime (7, 84).

## Disease Associations

HPgV-1 infection has been studied in a wide variety of people with and without chronic diseases. Taken together, studies do not show that the virus is clearly responsible for any acute or chronic disease (1, 28, 95, 98–105). Specifically, HPgV-1 is not associated with more extensive HCV-related disease, post-transplant liver disease or non-A to non-E hepatitis. This appears to be related to viral tropism, as evidence strongly suggests that the virus is lymphotropic. Supporting this hypothesis, removal of the donor liver in HCV/HPgV-1 co-infected individuals undergoing liver transplant results in a transient, but profound reduction in HCV viral load with no significant change in quantitative HPgV-1 viremia (106). HPgV-1 RNA is found in T bone marrow and spleen tissues (107–109), and in circulating T and B lymphocytes, monocytes, and NK cells (94, 110–116). Epidemiological studies also suggest that HPgV-1 is lymphotropic, and viral transmission mimics that of HIV (21, 76, 91, 94, 97, 117, 118).

Incubation of HPgV-1 with healthy donor peripheral blood mononuclear cells (PBMCs) or culture of PBMCs from HPgV-1 viremic individuals results in low level release of viral RNA into culture supernatant fluids, and T cell and NK cell functions are altered *in vitro* and *in vivo* (see below) (111–115, 119–121). The virus cannot be passaged for more than a few cycles in cell culture, and thus circulating PBMCs do not appear highly permissive for infection (94, 113). The primary cell type infected by HPgV-1 has not been identified, and there is no animal model of HPgV-1 infection to address this question directly. A *pegivirus* animal model was developed in rhesus macaques using a baboon pegivirus as the inoculum (42, 52). The distribution of baboon pegivirus RNA was consistent with studies of humans showing that bone marrow is the major location of both positive and negative (replicative) forms of HPgV-1 RNA (42, 109). Of note, this model may not be predictive of human pegivirus 1 infection, as there are sequence differences between simian and human viruses in critical regions of the genome.

Nevertheless, the specific cell type that is primarily infected and produces HPgV-1 *in vivo* remains a key unanswered question in HPgV-1 biology. Given the bone marrow location and distribution of virus RNA in human PBMCs, it has been speculated that a hematopoietic stem cell is the primary site of infection, and that a key replication factor (receptor, transcription factor, etc.) necessary for efficient and productive infection is lost during cell differentiation. Nevertheless, high total body levels of virus are present in infected individuals, with average plasma viral loads ranging from 1 x 10^5^ genome equivalents/mL (GE/mL) to as high as 1 x 10^7^ GE/mL (67, 122). Of note, viral load is distributed in a bi-phasic pattern. Approximately two thirds of healthy blood donors have an average viral load of 5 x 10^6^ GE/mL while the remaining one third of individuals have a viral load of approximately 8 x 10^3^ GE/mL (67). The reason for this biphasic distribution in viral load is unclear but may relate to host specific immunity, or perhaps to prior infection with some existing adaptive immune responses. It would be interesting to follow these subjects longitudinally to see if clearance rates over time differ based on viral load.

A variety of diseases have been studied to determine if there is an association with HPgV-1, and there is no clear association with fulminant hepatitis (98, 123, 124), aplastic anemia (15, 125, 126), hepatocellular carcinoma (103, 127, 128), multiple sclerosis (MS) (129), porphyria cutanea tarda (130), oral lichen planus or oral carcinoma (131), and others reviewed in (26–28, 95). However, there are two clinical situations in which HPgV-1 is associated with disease, non-Hodgkin’s lymphoma (NHL) and sporadic encephalitis.

In several studies including a meta-analysis, HPgV-1 infection is associated with approximately a 2.8-fold increased risk of developing NHL (132–135). HPgV-1 has also been associated with sporadic encephalitis, and viral RNA and antigens were detected in post-mortem brain tissues from two people with this condition (136, 137). A variant of HPgV-1 containing a deletion in the NS2 coding region was identified in the brain tissue of one subject, and this NS2 deletion virus appeared to replicate better in astrocytes than the wild type HPgV-1. HPgV-1 replication has recently been demonstrated in human astrocytes and microglia *in vitro*, though replication was not identified in neurons or an oligodendrocyte-derived cell line (137). HPgV-1 infection of human glia cells led to the suppression of peroxisome-associated gene expression which was accompanied by reduced expression of interferon-beta, interferon regulatory transcription factors 1 and 3, and mitochondrial antiviral signaling protein. These changes were consistent with findings in the brain tissue obtained from the patients with HPgV-1 and sporadic encephalitis (137). Given the high prevalence of HPgV, the high serum and PBMC viral load, and low rates of sporadic encephalitis, it remains unclear if HPgV-1 commonly infects neural tissue or has any direct neurological outcomes in those without encephalitis. Of note, in the rhesus model of *pegivirus* infection neural tissues did not demonstrate increased levels of viral RNA (42). It will be important to clarify the host feature(s) associated with putative HPgV-1-related encephalitis.

Among pegivirus infections in non-human species, no definitive association has been identified with a specific disease entity. Early reports suggested that a divergent member of the *pegivirus* genus found in horses (called Theiler’s disease-associated virus) was responsible for Theiler’s disease (44, 138). However, this conclusion was later shown to be spurious (139, 140). More recently, a novel pegivirus was identified in a research colony of common marmosets (Callithrix jacchus) in animals that died of lymphocytic enterocolitis (34). The investigators named this new virus the “southwest bike trail virus” (SOBV). Although identified in ill marmosets, a prospective analysis of a colony of 85 common marmosets failed to identify an association between SOBV infection and lymphocytic enterocolitis or any other disease. The prevalence of SOBV in common marmoset was high (34%) illustrating the difficulty in assigning disease causality for a prevalent virus (34). To date, SOBV has not been identified in common marmosets found in the wild and it is not clear if this species is the natural host for SOBV (34, 141).

## Clinical Evidence of Viral Interference

In the first 5 years following the discovery of HPgV, more than 600 papers were published examining the potential clinical outcomes of infection, with most of these studies conducted by research groups whose focus was on viral hepatitis. Once there was clear evidence that HPgV-1 does not cause acute or chronic liver disease (26, 27), research interest and publication rates fell rapidly. However, small studies from Japan, Germany, the U.S. and France described the role of HPgV-1 in liver disease in HIV coinfection (76, 83, 142, 143). These reports confirmed that HPgV-1 was not associated with liver disease; however, people with HIV and HPgV-1 coinfection appeared to have prolonged survival compared to those with HIV alone. These data were subsequently confirmed in two large, cross-sectional studies of people with HIV infection. The relative risk of mortality was 3.7-fold (95% C.I. 2.5 – 5.4) higher among those without HPgV-1 infection in a U.S. study (94) and also significantly higher in a German study (122), although relative hazard rates and confidence intervals were not described for the latter. Interestingly, HPgV-1 viral loads were inversely related to HIV RNA levels, and HPgV-1 infection did not prevent CD4 depletion (67, 94, 122, 144). Nevertheless, CD4 counts were higher overall in those with HPgV-1 compared to those uninfected in several studies (67, 76, 94, 122, 145, 146).

A legitimate criticism of these early studies related to the fact that the duration of HIV infection was unknown at the time of HPgV-1 sampling. If HPgV-1 were acquired late in infection this would bias prevalence towards those living longer with HIV (118). An alternative explanation for the beneficial observation suggested that HPgV, increasingly recognized as a lymphotropic virus, was simply a marker of conserved CD4 cells. If so, CD4 depletion accompanying HIV infection would lead to a loss of HPgV-1 viremia (Van der Bij et al., 2005). This latter explanation was not consistent with the finding that survival was reduced in PLWH who presented with very low CD4 counts (< 50/mm^3^) (94, 147).

To address these limitations, two studies were conducted in HIV cohorts with known or an estimated time of HIV infection. In the Multicenter AIDS Cohort Study group, HPgV-1 viremia and E2 antibody were characterized in 271 individuals with documented HIV seroconversion within 12 months of the first positive HIV antibody test. In this “early” group, no survival benefit was observed; however, follow up testing 5-6 years following seroconversion found that those without HPgV-1 infection were 2.83-fold more likely to die in subsequent observation compared to those who were persistently HPgV-1 viremic (29). In addition, almost 20% of HPgV-1 viremic individuals cleared HPgV-1 in the first 5 to 6 years after seroconversion. Individuals who cleared viremia had significantly higher mortality than those who were persistently viremic (3.85-fold) suggesting either that HPgV-1 clearance results in the loss of a beneficial factor, or that clearance of viremia is related to a host variable that overcomes HPgV-1 immune escape mechanisms and which hastens HIV-related disease. Two additional studies confirmed that loss of HPgV-1 viremia was associated with significantly increased mortality (118, 148).

The Amsterdam HIV Study Group also examined the role of HPgV-1 on survival in people living with HIV. Although their data were highly consistent with the MACS study, the authors applied several statistical models to the data and came to different conclusions (118). Similar to the MACS study, the primary, unadjusted analysis found that mortality was significantly lower (RH 0.52, 95% CI 0.32-0.85) in those with persistent HPgV-1 infection. Three additional statistical models were applied to these data. In the first model, which was controlled for age at seroconversion, antiretroviral therapy, CCR5 genotype, baseline CD4, and HIV RNA 1 year after seroconversion, persistent HPgV-1 viremia was significantly associated with reduced mortality (RH 0.57, 95% CI 0.35-0.94). In the second model, the same variables plus time-varying HIV viral load were controlled and there remained a survival benefit (relative hazard of mortality 0.47, 95% CI 0.35 – 0.93) in those with persistent HPgV-1 infection. In the third model, time-varying CD4 count was added to the other cotrolled variables, and there remained a survival benefit (RH mortality 0.74, 95% CI 0.45 – 1.25); however, statistical significance was lost. The authors concluded from these data that HPgV-1 viremia is a marker of CD4 T cell number and that HPgV-1 is not a causative factor in the improved survival observed in the other models (118).

Limitations of the Amsterdam study include that the date of seroconversion was calculated and not measured directly. Since the calculated method of HIV infection has a relatively wide time window for seroconversion (149), the duration of HIV infection in this group was far less accurate than in the MACS study. Secondly, several studies demonstrate that CD4 decline is slowed in HPgV-1 infected PLWH (29, 76, 94, 122, 145). Since death in HIV-1 infection is caused by immune deficiency directly correlated to CD4 depletion, CD4 count is in the causal pathway for death. Controlling for time-varying CD4 removes the differences in CD4 decline in those with and without HPgV-1 infection, and from a statistical standpoint, controlling avariable in the causal pathway should remove any potential benefit of HPgV-1 (147). Nevertheless, there remained a 26% reduction in mortality in those with persistent HPgV in this model. This suggests that pegivirus viremia influences mortality by more than expected from its altered rate of CD4 depletion (147, 150). A meta-analysis of published studies meeting rigorous criteria confirmed a reduction in mortality in HIV infection for those with HPgV-1 co-infection (RH 0.41) (96).

Despite a large number of studies showing survival advantage, lower HIV VL, and higher CD4 in those with HIV and HPgV-1 coinfection, the interpretation of the Amsterdam data continues to reduce acceptance of the apparent beneficial effect of HPgV-1 on HIV survival. Since there is not a good animal model for HPgV, and human challenge experiments proposed to the FDA were not allowed to proceed (using licensed blood products), Koch’s postulates have not been confirmed. A close proxy to Koch’s postulates was assessed by Vahidnia et al., who tested plasma samples obtained from people living with HIV who had received blood transfusions in the 1980s and 1990s. Pre- and post-transfusion samples and samples from the transfused blood product were available for HPgV-1 viremia testing, and individuals who acquired HPgV-1 from transfusion were found to have a reduced risk of death (RH 0.22) compared to those who did not acquire HPgV-1 (151), confirming a direct role for HPgV-1 infection and prolonged survival in HIV infection.

Finally, two studies found that HPgV-1 infection in women is associated with reduced HIV transmission from mother to child in the pre-highly active antiretroviral therapy era. A U.S. study found an association between maternal HPgV-1 viremia in the third trimester and reduced HIV infection in the child (152). A large study in Thailand found that, among infants who acquired HPgV-1 during delivery, HIV transmission was reduced by 87%, further supporting an HPgV-related reductions in HIV disease outcomes (78).

Taken together, the data demonstrating a survival benefit among PLWH who have HPgV-1 coinfection is thus supported by numerous clinical studies. Two papers recommend studying HPgV-1 infection as a “biotherapy” for HIV infection (153, 154). To date, regulatory groups have not approved this approach. The following section will summarize data investigating mechanisms by which HPgV-1 might influence HIV disease outcomes.

## Potential Mechanisms of HPgV-1 Interference With HIV

### Evidence of a Direct Antiviral Effect

Infection of healthy human PBMCs with HPgV-1 for 24 hours prior to adding HIV results in more than a 90% reduction in HIV production (94, 119, 155). The extent of inhibition in HIV replication was reduced if HPgV-1 was added simultaneously or 24 hours after HIV infection, although HIV replication was still significantly reduced (94). Of note, PBMC infection with HPgV-1 is inefficient, and replication kinetics are extremely virus- and host-cell specific (113). Maintaining fresh PBMCs obtained from infected individuals in cell culture leads to low levels of HPgV-1 production over long periods of time, up to 10 weeks in many instances (114, 115). In this approach, the majority of PBMCs die in the first week but a small population of minimally metabolically active lymphocytes persist and release low levels of HPgV-1 RNA into the culture supernatants. Early studies found that expression of the HIV co-receptor CCR5 was reduced on CD4+ and CD8+ T cells in individuals with HPgV-1 and HIV co-infection compared to HIV mono-infected (156, 157). This result was recapitulated and extended *in vitro* (155), and incubation of PBMCs with HPgV-1 upregulated chemokine ligands for the two HIV co-receptors, CCR5 and CXCR4. Further, co-receptor expression was reduced under these conditions (155, 158).

**Figure 3:**
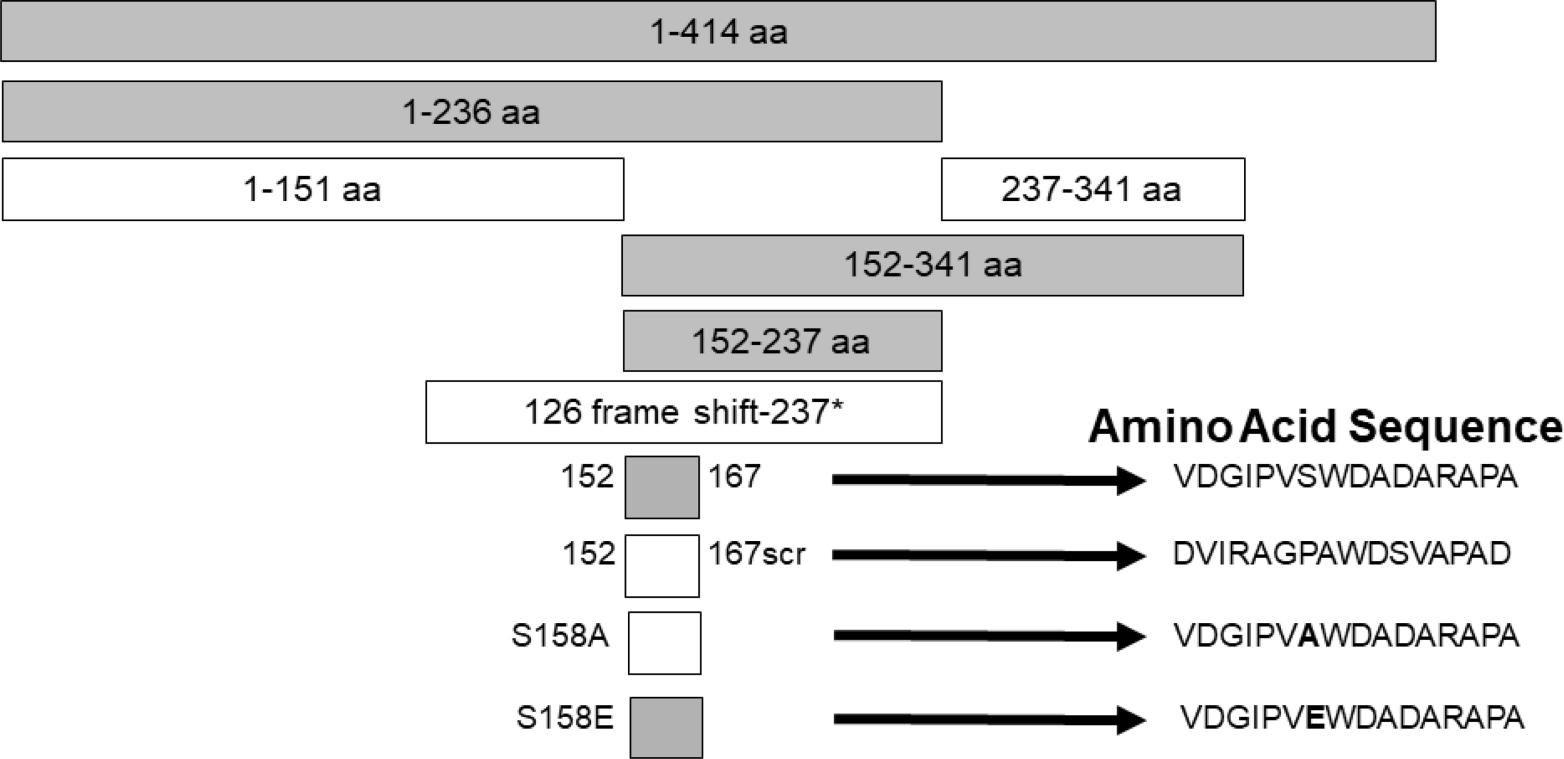
Expression of HPgV NS5A peptides inhibits HIV replication, adapted from References (159, 162). Schematic representation of the region of NS5A protein stably expressed in CD4+ T cells. The regions that inhibited HIV replication are shaded. Full-length NS5A protein (414 amino acids) is shown on top, and deletion proteins below. The 16 aa peptide from 152 – 167 was sufficient to inhibit HIV replication. Scrambling the order of the 16 amino acids or mutating the putative phosphorylation site (serine) at amino acid 158 abolished HIV inhibition (scr), while mutating this to a glutamic acid (phosphomimetic) restored HIV inhibition functions. FS represents the full-length RNA for NS5A in which a frame shift insertion was created to abolish NS5A protein expression.

Studies were designed to examine the ability of specific viral components to inhibit HIV with the hope of identifying novel antiviral agents that may have low risk of mutational escape. Expression of the NS5A protein in a CD4+ T cell line resulted in highly (>90%) suppressed HIV inhibition (159, 160). By making expressing a series of NS5A deletions, the inhibitory region was found to reside in a small (16 amino acid) E2 peptide region. HIV inhibition was observed with viruses representing different genotypes, and NS5A reduced CD4 and CXCR4 expression and increased production of the CXCR4 ligand SDF-1 (159, 161, 162). HIV inhibition required expression of the NS5A peptide region, as cells expressing NS5A coding RNA with a frameshift inserted to abolish protein synthesis did not inhibit HIV. (159) (**Figure 3**).

In addition to NS5A, recombinant HPgV-1 envelope (E2) protein inhibited HIV replication *in vitro* (158, 163, 164). Similar to the approach used with NS5A, expression of HPgV-1 E2 in CD4+ T cell lines also potently inhibited HIV replication. Deletion mutagenesis identified a 17 amino acid E2 peptide previously shown to be involved in virus cellular binding was sufficient to inhibit HIV replication (McLinden et al., 2006;^164^, ^165^). As with NS5A, point mutations in this E2 peptide reversed the inhibition, and synthetic peptides containing the HIV TAT transduction domain peptide to provide entry into cells resulted in HIV inhibition compared to control peptides (164). Additional peptide regions of HPgV-1 E2 or E1 interfere with HIV replication (164, 166–174). Of note, E2 peptide regions shown to inhibit HIV *in vitro* are not consistent between different experimental systems; however, HPgV-1 E2- and E1-mediated HIV inhibition correlated with reduced HIV entry in most reports (163, 164, 166, 169–173).

In addition to reducing HIV entry, HPgV-1 E2 protein appears to have additional effects on HIV replication by inhibiting HIV GAG assembly and release (175). Specifically, expression of HPgV-1 E2 inhibited Gag processing with no change in the GAG precursor Pr55, but with reduced levels of the capsid regions CA24 and MAp17, both of which are phosphorylated motifs (175, 176). Glycosylated HPgV-1 E2 disrupted GAG trafficking to the plasma membrane, and reduced HIV particle production (175). Taken together HPgV-1 E2 protein and possibly an HPgV-1 fusion motif on E1 inhibits HIV replication *in vitro*.

The cellular receptor for HPgV-1 remains uncharacterized. An early study suggested that HPgV-1 E2 binds to CD81 (177), which serves as the major HCV E2 binding receptor on cells (178, 179), However, subsequent studies using CD81 deificient cell lines and competition experiments showed that CD81 is not required for E2 cell binding (180). Although these finding suggest that potential, peptide-based or small molecule HIV inhibitors based on HPgV-1 peptides might be developed, the widespread use of highly active, combination antiretroviral therapies essentially removed the need to pursue this novel therapeutic approach.

A third HPgV-1 protein has antiviral activity *in vitro*. The HPgV-1 NS3 serine protease inhibits HIV replication *in vitro*, and this inhibition requires protease activity (72). Further, HPgV-1 NS3 protease inhibits the induction of host Type 1 interferon responses independent of its antiviral activity (181). Type 1 interferons are important contributors to a complex, cross-regulatory immune network, thus HPgV-1 interference with this network may enhance viral replication by reducing innate immune responses, or be antiviral by reducing host immune activation, a key element in HIV-1 pathogenesis (182).While HPgV-1 viremia is associated with the greatest reduction in mortality in studies of people living with HIV (PLWH), two studies identified an association between the presence of HPgV-1 E2 antibody and improved survival in those without viremia or E2 antibody (29, 91). Characterization of HPgV-1 E2 antigenicity is limited, although anti-peptide antibodies were detected directed against inconsistent regions of the E2 protein (183, 184). These studies do not contain controls with and without HPgV-1 viremia, or pre- or post-HPgV-1 infection, and it is not clear that these tests can differentiate active or cleared viremia, thus their utility in diagnostic assays is untested.

Limited numbers of well characterized E2 monoclonal antibodies (MAbs) have been generated (185), and one of these antibodies reacts with the same 17 amino acid peptide region responsible for HIV inhibition in cell culture systems (164, 186). Seven of the eight human MAbs generated by DNA immunization recognize conformational epitopes on E2, and based on competition studies, bind to clustered and overlapping epitopes identifying a putative single, immunodominant antigenic site on the virus particle (185, 186). The one antibody that bound a linear epitope reacted with two overlapping peptides. Surprisingly the antibody did not bind to the 9 amino acid overlap domain and additional amino acids from either the N-terminus or C-terminus of this overlap was required for antibody binding, suggesting a conformational component to the antibody binding site (186).

Since two clinical studies found an association between HPgV-1 E2 antibodies and improved survival, HPgV-1 E2 MAbs were incubated with HIV and surprisingly these neutralized HIV infection (165). Rabbit and murine sera post-E2 immunization also neutralized HIV infectivity and precipitated HIV particles from diverse HIV lineages while pre-immunization sera did not (165). Thus, HPgV-1 E2 antibodies raised against recombinant protein or peptides have neutralizing activity against HIV and appear to react with non-gp120 regions of the HIV envelope (165). HPgV-1 E2 protein was included in a multivalent HIV vaccine candidate that shows promise against infection; however, the relative importance of this heterologous antigen remains to be elucidated (187).

### Clinical and Laboratory Evidence for HPgV-1 Immune Modulation

Several lines of clinical evidence emerged suggesting that HPgV-1 modulates host immune responses, and that these immunomodulatory consequences of infection may influence HIV pathogenesis. Early studies found reduced levels of CCR5 levels on CD4+ and CD8+ T cells in HPgV-HIV co-infected individuals compared to those without HPgV-1 (156, 157, 188, 189). This was initially considered an antiviral effect reducing HIV entry; however, CCR5 is upregulated by immune activation, a key requirement for HIV replication and pathogenesis (190–193). As noted above, *in vitro* studies resulted in reduced CCR5 and or CXCR4 levels and upregulation of inhibitory chemokine ligands (155, 158, 159). The effect of HPgV-1 on HIV reactivation was evaluated using PBMCs obtained from PLWH who were virally suppressed for more than 6 months. HPgV-1 reduced IL-2 and PHA stimulated latent HIV reactivation *in vitro*, suggesting that HPgV-1 may hinder activation-induced therapeutic “cure” strategies for HIV, especially since up to 41% of PLWH HPgV-1 coinfection (84, 114, 194).

Several clinical studies suggest that HPgV-1 reduces immune activation. For example, longitudinal measurement of circulating cytokine levels in HIV infected individuals found that HPgV-1 coinfection is associated with maintenance of a Th1 profile while those with mono-infection become polarized towards a Th2 profile (145). This is supported *in vitro* as NS5A expression in a CD4+ T cell line downregulated CD4 and key Th2 cytokines (e.g. IL13) (195). Some data suggest that HPgV-1 genotypic differences may alter cytokine levels (196).

Recombinant IL-2 was tested as an adjunctive therapy for HIV in the 1990s (197). Although IL-2 therapy resulted in highly significant increases in CD4 counts, clinical endpoints were not improved and therapy had many side effects and was never pursued for clinical use (197). To assess the potential relationship between HPgV-1 infection and IL-2-mediated CD4 expansion, HPgV-1 RNA was characterized in samples obtained from HIV-positive subjects prospectively enrolled in a randomized trial of IL-2. Surprisingly, CD4 counts did not increase in those with HPgV-1 co-infection whereas those without HPgV-1 had a rise of more than 1,000 CD4 cells/mm^3^, suggesting that HPgV-1 interferes with CD4 expansion and IL-2 signaling (198). Examining this *in vitro*, recombinant HPgV-1 E2 protein reduced IL-2 receptor mediated STAT5 activation following IL-2 receptor stimulation (199). Further, HPgV-1 E2 protein reduced T cell proliferation and cell surface markers of T cell activation following stimulation through the T cell receptor (TCR) (121, 199).

Maidana-Giret et al. found that HPgV-1 was associated with reduced CD4+ and CD8+ T cell activation markers (CD38, HLA DR) during acute HIV infection (188). This was confirmed in individuals with chronic HIV infection, with HPgV-1 co-infected people having significantly reduced activation marker expression regardless of viral suppression, and also with reduced frequency of circulating double negative T cells (189, 200). Immune activation markers were not only reduced in T cells, as HPgV-1 coinfection was associated with reduced activation markers on B cells, monocytes, and NK cells (201). Since HIV induces chronic immune activation, and increased activation markers are highly predictive of HIV disease progression, these studies suggest that HPgV-1 mediates its associated survival benefit by dampening immune activation (202).

**Figure 4:**
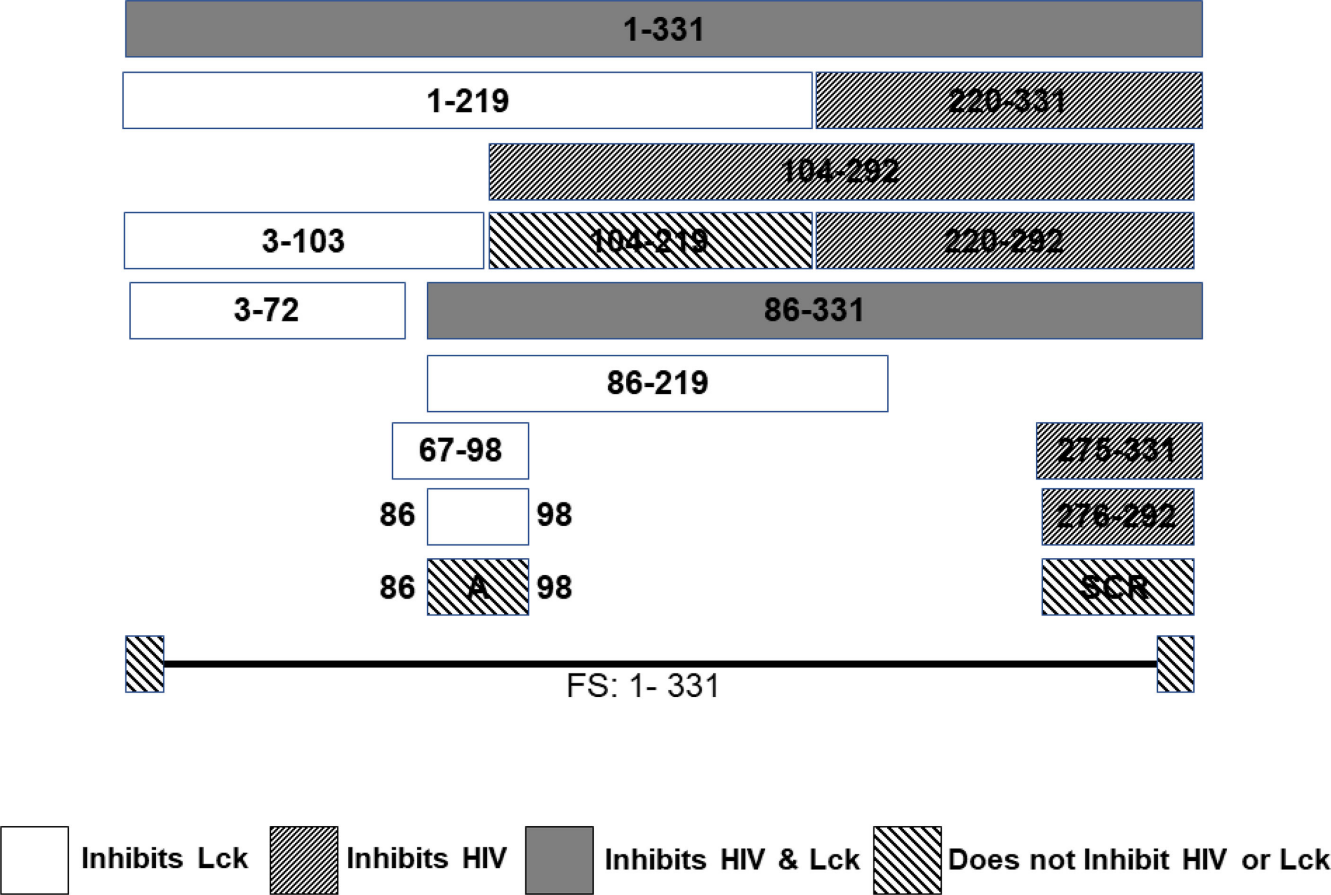
HPgV E2 protein has two discrete regions that interfere with either HIV replication or T cell activation adapted from References (121, 164). Schematic representation of the regions of E2 protein expressed by stably transfected CD4+ T cells that either inhibit HIV replication or blunts T cell receptor (TCR) signaling. HPgV E2 regions that both inhibited HIV and TCR signaling are shaded. Those that only interfere with TCR are white. E2 regions inhibiting HIV replication have narrow, forward slash shading, while regions that did not interfere with either HIV replication or TCR signaling are wide, backslash shaded. Full-length HPgV E2 protein with the C terminal transmembrane domain deleted (331 amino acids) is shown on top, and deletion proteins below. Scrambling the order of the 17 amino acids interfering with HIV replication abolished HIV inhibition. Mutation of a tyrosine in the TCR inhibitory peptide to an alanine abolished TCR inhibition. FS represents the full-length RNA for E2 in which a frame shift insertion was created to abolish E2 protein expression.

A series of CD4+ T cell lines expressing E2 protein or E2 protein deletions identified a 13 amino acid peptide region that was sufficient to reduce TCR-mediated activation (121). HPgV-1 reduced activation of the proximal tyrosine Src kinase (LCK) in the TCR-induced signaling cascade, and the region involved was independent of the peptide region that inhibited HIV replication (121, 164) (**Figure 4**). The E2 amino acid sequence required for TCR signaling inhibition is predicted to be a substrate for LCK, and mutation of the tyrosine in this peptide restored TCR function. Further, HPgV-1 E2 was phosphorylated by LCK *in vitro*, suggesting that E2 competitively inhibits TCR-mediated T cell activation and IL-2 release (121, 199). In addition, IL-12 receptor-mediated NK cell activation is reduced by HPgV-1 and recombinant E2 (203), consistent with clinical observations that NK cell activation is reduced in HPgV-HIV coinfected individuals compared to those with HIV monoinfection (201).

Of note, TCR signaling is not abolished by HPgV-1 or E2, and increasing the stimulus overcomes the inhibition *in vitro*. This is consistent with the finding that people who have HPgV_1 infection are not clinically immune compromised. Further, since HIV inhibition and TCR interference require different regions of the E2 protein, it suggests that both antiviral and reduced T cell activation influence HIV replication.

The innate antiviral immune response influences HIV disease progression, so potentially HPgV-1 might activate innate immunity in PLWH and thus slow disease progression. Lalle et al. found enhanced activation of the interferon system and circulating dendritic cells including promotion of interferon-gamma and activation and maturation of circulating dendritic cells in HPgV-1 co-infected individuals compared to HIV-mono infection (204). In contrast, Hoseini et al. did not find any differences in interferon producing cells among HPgV-1 – HIV coinfected individuals (205), and serum cytokines revealed a reduction in proinflammatory cytokines one year after HPgV-1 infection, although in the acute phase of HPgV-1 (first three months after infection), pro-apoptotic cytokines were upregulated (206). Chronic HPgV-1 coinfection in HIV was associated with significantly lower numbers of Fas-expressing CD4+ T cells when compared to HIV mono-infected (207). Since Fas expression correlates with Fas-mediated apoptosis, HPgV-1 may potentially slow CD4+ depletion by reducing apoptosis and T cell activation (115, 116, 156, 157, 188, 189, 199, 200, 203, 207).

Like HCV, HPgV-1 viremia can be cleared in individuals treated with alpha-interferon (208–212), though factors predicting interferon susceptibility have not been identified. Like HCV, sequence polymorphisms in the HPgV-1 NS5A protein correlate with interferon sensitivity, (213–215). Two HPgV-1 proteins have direct anti-interferon activities. NS5A blocks dimerization of PKR following interferon stimulation (215), and as noted above, the NS3 serine protease blocks the induction of Type 1 interferons and this requires active HPgV-1 protease activity (181).

**Figure 5:**
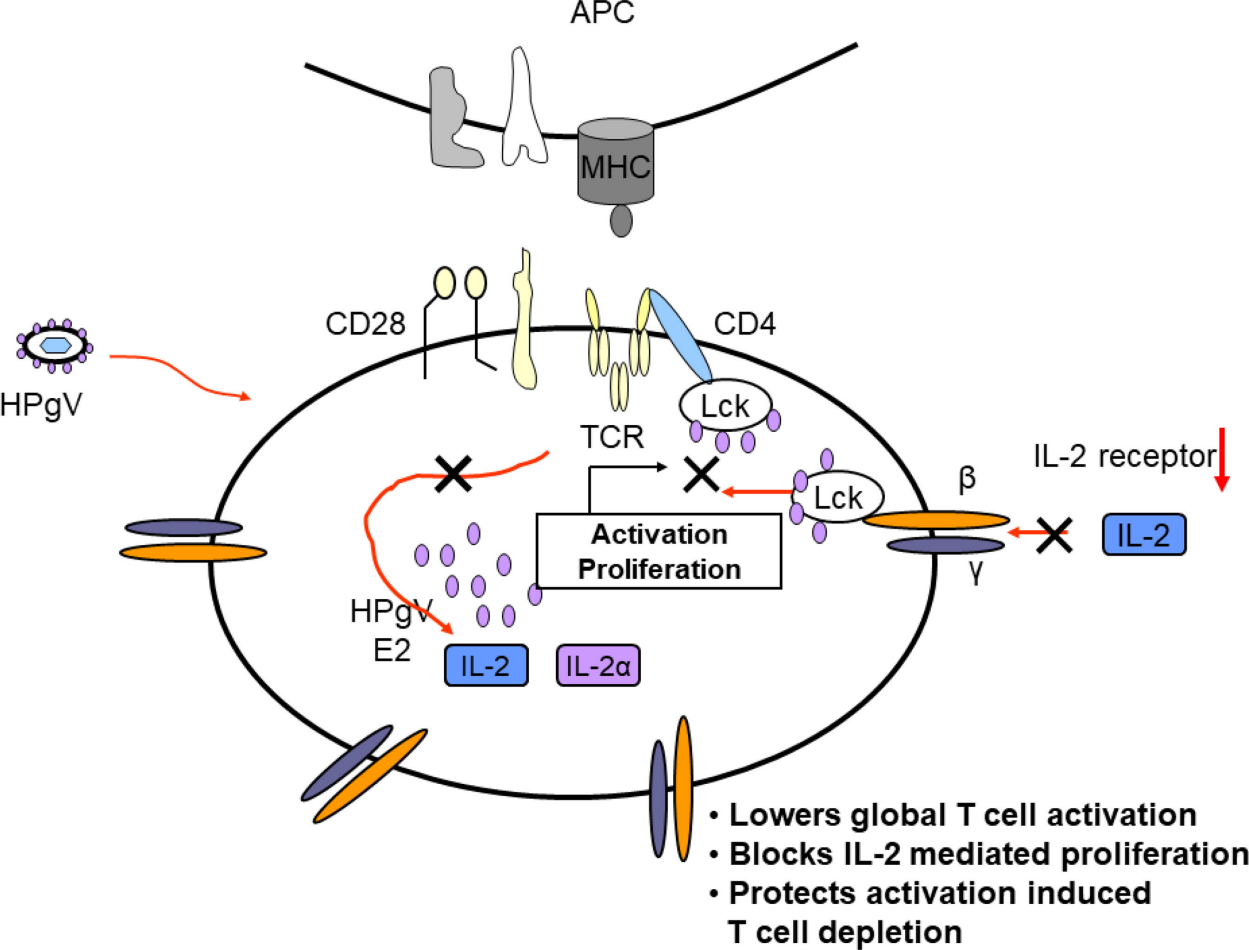
Proposed model of mechanisms by which HPgV interferes with T cell activation, proliferation, and depletion. Virus particles containing HPgV E2 protein interact with T cell and other immune cells and genomic RNA is translated in the cytoplasm, producing additional E2 protein. The effects of HPgV particles and/or intracellular E2 proteins interfere with T cell receptor-mediated activation of Lck, reducing cellular IL-2 release. HPgV also interferes with IL-2 receptor function further reducing T cell activation and proliferation. These effects can be overcome with sufficient TCR stimulus.

Taken together, clinical studies illustrate that HPgV-1 infection is associated with reduced immune activation despite the presence of high viral loads, and laboratory studies identify several mechanisms by which HPgV-1 interferes with immune cell functions. Mechanisms supported by *in vitro* or *in vivo* evidence are summarized in **Figure 5**. Since HPgV-1 is not associated with immune suppression, the effects of HPgV-1 appear to reduce but not block immunologic hyper-activation. Since HIV disease progression is largely driven by chronic immune activation, it is likely that these immunologic effects of HPgV-1 contribute to the beneficial outcomes observed in numerous clinical studies. Since immune activation can be stimulated with sufficient stimulus, HPgV-1 appears to provide a beneficial dampening of immune activation without resultant immune deficiency.

## HPgV-1 Influences Other Immune-Mediated Disease States

Viral-mediated interference with T cell activation may be a common feature of viral immune modulation (Stapleton JT, 2014), and in addition to HIV-1, there are at least three additional diseases in which HPgV-1 infection is association with beneficial clinical outcomes. Although studies of HCV and HPgV-1 did not identify synergistic pathology, a beneficial role for HPgV-1 infection was found in individuals with triple infection with HIV, HCV and HPgV-1 compared to those with HIV and HCV coinfection (216). Specifically, HPgV-1 infection was associated with slower progression of HCV-related liver disease, and also with reduced LCK activation in intrahepatic T cells (216, 217). Since HCV pathogenesis is driven by immunologic destruction of infected hepatocytes, reducing T cell activation and proliferation likely contributes to the improved outcomes in HCV-induced liver disease (24, 218).

Similarly, Ebola virus infection is associated with cytokine storm and high mortality rates (219). Studies in West Africans with Ebola found that mortality in those with HPgV-1 coinfection was 47% compared to 78% in those without HPgV-1 coinfection, presumably due to the effects of HPgV-1 on immune activation (219). Although there have not been studies reported to date to address this, it would be of interest to see if primary COVID-19 outcomes are ameliorated by HPgV-1 infection since severe SARS-CoV-2 infection is associated with cytokine storm (220).

One additional immune-mediated clinical disease may be influenced by HPgV-1 infection. Vu et al. found that, following stem cell transplantation, both the rate of severe graft-versus-host-disease and overall mortality were reduced in individuals with HPgV-1 infection (221). This finding requires confirmation, but given the effects of HPgV-1 on immune activation, there is biologic plausibility supporting a beneficial interaction (222).

## Conclusions and Key Questions Regarding HPgV

HPgV-1 is associated with an increased risk of lymphoma and possibly with sporadic encephalitis. However, given the high prevalence of infection and lack of commonly recognized disease states, in concert with the finding that persistent infection is beneficial in HIV coinfected individuals (and possibly other disease states), HPgV appears to represent a largely symbiotic infection within the human virome that has little or no pathogenicity. Several direct anti-retroviral actions of HPgV-1 have been identified, and these likely contribute to the improved survival observed in people living with HIV. Examination of the structural components of anti-HIV peptides may facilitate the development of cellular-based, small molecule HIV inhibitors, and identifying the cross-reactive HIV antigen(s) recognized by HPgV-1 E2 antibody may facilitate development of HIV vaccines (159, 164, 165, 168, 169, 171, 173).

In addition to developing HIV therapeutics, HPgV-1 is highly associated with immune modulation of human T cells, B cells, NK cells and monocytes, suggesting that this aspect of the virus provides beneficial, anti-inflammatory effects without inducing clinically evident immune compromise (188, 189, 201). In persistently viremic individuals, high rates of viral replication are observed concurrently with reduced markers of immune activation and in most, no detectable anti-HPgV-1 antibodies detected (7).

As a relatively non-pathogenic, lymphotropic virus that infects and preferentially replicates in bone marrow and spleen, HPgV-1 potentially could be used as either a novel gene therapy delivery system or as a bio-vaccine for HIV (153, 154). Further study of the role of HPgV infection on disease outcomes in immune-mediated diseases could provide insights into the natural history of many immune-mediated diseases, including most recently COVID-19. The effect, if any of HPgV-1 on SARS-CoV-2 infection has not been examined at the time of writing.

Currently, the biggest problem hindering HPgV-1 development for therapeutic use relates to the difficulties in growing the virus *in vitro*. While virus replicates in lymphocytes, NK cells, and neural cells *in vitro* (110–113, 115, 116, 120, 137, 203), infection cannot be maintained in cell culture for more than handful of passages, suggesting either abortive infection or slow infection with dilutional loss of infected cells. Specifically, most of the cells in PBMC cultures obtained from HPgV-1 infected individuals die rapidly, leaving a small subset of primary mononuclear cells that survive for weeks to months producing low levels of virus (115). In CD4+ Jurkat T cell lines, intracellular viral RNA and antigens are detected following infection (113, 119); however, the amount virus released slowly decays, and after several passes of supernatant fluids to new, uninfected cells, the infection is lost. Since HPgV-1 slows cellular proliferation induced by IL-2 or TCR-stimulation (121, 199), this may reflect a virus-driven interference with Src kinase-mediated cellular proliferation, resulting in gradual loss of infected cells that produce such low levels of virus that infection of new cells is gradually lost.

Many approaches to identify specific cell types that support HPgV-1 replication have been attempted including use of CD34+ human stem cells, interferon-resistant cells, and other innate knockout cell lines without success (116). The recent finding that goose pegivirus replicates well in embryonated goose eggs and primary goose embryo fibroblasts raises hope that novel culture systems can be developed for HPgV-1 (55).

Another important limitation in HPgV-1 research is the validated association of HPgV-1 infection with the development of lymphoma and the recent suggestion of an association with encephalitis. Given the effects of HPgV-1 on immune activation, the most logical explanation for increased risk of lymphoma relates to a reduction in immune surveillance, though a direct causal effect on lymphoma needs to be excluded (133–135). The two to three-fold increase in risk of lymphoma in HPgV-1 viremic individuals is significant, but does not explain the vast majority of lymphoma cases. Further, the age of HPgV-1 infection (sexually active adolescents to late-mid life) is the opposite that of maximal lymphoma risk (223).

In summary, HPgV-1 is a highly prevalent, low- or non-virulent human infection that reaches high viral loads in plasma and is associate with reductions in human immune activation markers. *Pegiviruses* are increasingly being identified in diverse hosts, and their apparent longevity in humans and non-human primates suggests that they convey an overarching beneficial effect on health. Future studies to determine the breadth and magnitude of these effects are warranted.

## Author Contributions

The author confirms being the sole contributor of this work and has approved it for publication.

## Funding

This study was supported in part by a Veterans Administration Merit Review grant (BX000207).

## Conflict of Interest

The author JS has patents related to the use of GB virus C in the potential treatment of HIV-1 infection.

## Publisher’s Note

All claims expressed in this article are solely those of the authors and do not necessarily represent those of their affiliated organizations, or those of the publisher, the editors and the reviewers. Any product that may be evaluated in this article, or claim that may be made by its manufacturer, is not guaranteed or endorsed by the publisher.
